# Pre-test probability for SARS-Cov-2-related infection score: The PARIS score

**DOI:** 10.1371/journal.pone.0243342

**Published:** 2020-12-17

**Authors:** Mickael Tordjman, Ahmed Mekki, Rahul D. Mali, Ines Saab, Guillaume Chassagnon, Enora Guillo, Robert Burns, Deborah Eshagh, Sebastien Beaune, Guillaume Madelin, Simon Bessis, Antoine Feydy, Fadila Mihoubi, Benoit Doumenc, Luc Mouthon, Robert-Yves Carlier, Jean-Luc Drapé, Marie-Pierre Revel

**Affiliations:** 1 Department of Radiology, Cochin Hospital, APHP, Paris, France; 2 Department of Radiology, Ambroise Paré Hospital, APHP, Boulogne, France; 3 Center for Biomedical Imaging, Department of Radiology, New York University School of Medicine, New York, New York, United States of America; 4 Université de Paris, Paris, France; 5 Department of Internal Medicine, Saint Antoine Hospital, APHP, Paris, France; 6 Emergency Department, Ambroise Paré Hospital, APHP, Boulogne, France; 7 Department of Infectious diseases, Raymond Poincaré Hospital, APHP, Garches, France; 8 Emergency Department, Cochin Hospital, APHP, Paris, France; 9 Department of Internal Medicine, Cochin Hospital, APHP, Paris, France; 10 Department of Radiology, Raymond Poincaré Hospital, APHP, Garches, France; 11 DMU Smart Imaging, APHP, Paris, France; Fundacao Oswaldo Cruz, BRAZIL

## Abstract

**Introduction:**

In numerous countries, large population testing is impossible due to the limited availability of RT-PCR kits and CT-scans. This study aimed to determine a pre-test probability score for SARS-CoV-2 infection.

**Methods:**

This multicenter retrospective study (4 University Hospitals) included patients with clinical suspicion of SARS-CoV-2 infection. Demographic characteristics, clinical symptoms, and results of blood tests (complete white blood cell count, serum electrolytes and CRP) were collected. A pre-test probability score was derived from univariate analyses of clinical and biological variables between patients and controls, followed by multivariate binary logistic analysis to determine the independent variables associated with SARS-CoV-2 infection.

**Results:**

605 patients were included between March 10^th^ and April 30^th^, 2020 (200 patients for the training cohort, 405 consecutive patients for the validation cohort). In the multivariate analysis, lymphocyte (<1.3 G/L), eosinophil (<0.06 G/L), basophil (<0.04 G/L) and neutrophil counts (<5 G/L) were associated with high probability of SARS-CoV-2 infection but no clinical variable was statistically significant. The score had a good performance in the validation cohort (AUC = 0.918 (CI: [0.891–0.946]; STD = 0.014) with a Positive Predictive Value of high-probability score of 93% (95%CI: [0.89–0.96]). Furthermore, a low-probability score excluded SARS-CoV-2 infection with a Negative Predictive Value of 98% (95%CI: [0.93–0.99]). The performance of the score was stable even during the last period of the study (15-30^th^ April) with more controls than infected patients.

**Conclusions:**

The PARIS score has a good performance to categorize the pre-test probability of SARS-CoV-2 infection based on complete white blood cell count. It could help clinicians adapt testing and for rapid triage of patients before test results.

## Introduction

Coronavirus disease 2019 (Covid-19) is a major global threat that has already caused more than 500,000 deaths worldwide. Based on data from patients with laboratory-confirmed Covid-19 from mainland China, admission to intensive care unit (ICU), invasive mechanical ventilation or death occurred in 6.1% [1]. The case-fatality rates (CFR) vary in the different countries, with for example less than 0.5% in South Korea and Germany [2]. These variations might be due to comprehensive screening strategies in the countries with lower CFR values allowing the identification of a large number of individuals with mild symptoms and lower mortality risk, whereas only patients with severe symptoms are tested in others countries due to the lack of availability of RT-PCR kits to undertake large scale population testing [3].

Reverse Transcription Polymerase Chain Reaction (RT-PCR) is currently the test of reference to diagnose patients with SARS-CoV-2 infection [4]. Computed Tomography (CT)-scan has a reported high sensitivity [5] but radiological signs can be delayed after disease onset, with up to 56% CT negativity in the first 3 days of symptomatic infection [6]. Moreover, performing large scale CT scanning during the current pandemic is made difficult by the need to apply rigorous disinfection protocols between patients [7] without mentioning that it is problematic to induce radiation exposure for asymptomatic/paucisymptomatic patients. The Fleischner Society recently published a Multinational Consensus Statement on the use of thoracic imaging based on different scenarios [8]. Imaging is indicated in case of moderate-to-severe disease manifestations, but not indicated in case of mild symptoms consistent with COVID-19 and no risk factor for disease progression. Even though CT-scan has a high sensitivity (71–95% for PCR and 94–98% for CT), radiologists may still experience some difficulties in differentiating COVID-19 from non-COVID pneumonia [9–11]. False-negativity of PCR or CT as well as COVID-19 pneumonia mimickers on CT may lead to inaccurate diagnoses. Pre-test probability combining clinical and biological features has proven to be a very useful tool, already used in clinical practice for the management of patients with a suspicion of pulmonary embolism [12]. Therefore, we aimed to derive a new prediction score based on clinical and biological variables, independently from subjective clinical evaluation by assessing a retrospective cohort of patients admitted to the emergency department (ED) of our institution for a clinical suspicion of SARS-Cov-2 infection. We validated this **P**re-test probability for S**AR**S-Cov-2 **I**nfection based on **S**coring (PARIS score) in a distinct multicenter cohort of patients admitted for the same reason in ED or infectious disease departments of three other hospitals.

## Material and methods

This retrospective observational study has been approved by our local ethics committee (Institutional Review Board: “Comité local d’éthique pour les publications de l’hôpital Cochin” CLEP N° AAA-2020-08014), which waived the need for patient consent. Patients were recruited from the ED, internal medicine and infectious disease departments (consultations) of four different hospitals (Cochin Hospital, Paris; Hotel-Dieu Hospital, Paris; Ambroise Paré Hospital, Boulogne; Raymond Poincaré Hospital, Garches) between March 10^th^ and April 30^th^, 2020. The retrospective data used for this study were obtained between March 15^th^ and May 15^th^, 2020.

### Derivation cohort

We developed a diagnostic strategy for SARS-Cov-2 infection based on clinical and biological features to determine pre-test probability before RT-PCR and/or CT-scan. We recruited a retrospective cohort of outpatients that had both RT-PCR and CT-scan results available with a 1:1 patient:control inclusion ratio, who had been evaluated at the ED of Cochin Hospital (Paris, France) for a suspicion of SARS-Cov-2 infection between March 10^th^ and April 8^th^, 2020. Exclusion criteria were: 1) diagnosis still under investigation; 2) lack of blood test including complete white blood cell count and serum electrolytes; 3) absence of reported clinical characteristics. We chose to include one hundred consecutive patients with RT-PCR positivity and 100 consecutive controls in this cohort (controls were patients with clinical suspicion of SARS-Cov-2 for whom RT-PCR and CT were negative). Final diagnoses of controls are presented in S1 Table. Clinical examination was standardized at the ED. Demographic characteristics and comorbidities including hypertension, respiratory diseases (asthma, restrictive or chronic obstructive pulmonary disease (COPD)), immunodeficiency, renal insufficiency were recorded. Furthermore, clinical symptoms such as cough, fever, headache, diarrhea, anosmia, ageusia, oxygen desaturation were evaluated. Finally, biological tests including results of complete white blood cell count, serum electrolytes and C Reactive Protein (CRP) were analyzed. A final diagnosis of SARS-Cov-2 infection was retained if the patient had a confirmed diagnosis with positive RT-PCR and/or CT-scan showing signs of COVID-19 pneumonia (ground-glass opacities with subpleural predominance with or without focal consolidation). If the RT-PCR was negative, patients were considered as COVID+ if CT-scan images evaluated by a senior radiologist specialized in thoracic imaging (>10 years of experience) were typical for COVID-19 pneumonia. Thus, controls were patients with both negative RT-PCR results and negative CT-scans. The diagnoses of controls were recorded, but PCR tests of other viruses than SARS-Cov-2 (Influenza, Rhinovirus…) were not always performed (performed in only 10 of the 144 controls). We evaluated clinical and biological variables to find the ones associated with SARS-Cov-2 infection. We only included basic biological tests, widely available and affordable, which are adapted for a pre-test probability score.

### Validation cohort

Our pre-test diagnostic score was validated between March 10^th^ and April 30^th^, 2020 on 405 consecutive outpatients suspected of SARS-Cov-2 infection (different from those of the derivation cohort) with both RT-PCR and CT-scan results available. Patients were confirmed to have SARS-Cov-2 infection based on RT-PCR positivity. If the RT-PCR was negative, patients were considered as COVID+ in this cohort if CT-scan images evaluated by a senior radiologist specialized in thoracic imaging (>10 years of experience) were typical for COVID-19 pneumonia (N = 19). Patients with unavailability of initial complete white blood cell count were excluded.

### Statistical analysis

Univariate analyses were performed to select potential independent variables of SARS-Cov-2 infection to include in the multivariate analysis. The Mann Whitney U test was performed for comparison of continuous variables such as hemoglobin, and chi-square test was used for comparison of nominal variables. Exact p-values were computed for the Mann Whitney tests without any assumption of normality or asymptomatic approximation. A p-value less than 0.05 was considered as statistically significant (two-tailed). The statistically significant variables were evaluated to find the optimal cut-offs to differentiate patients and controls based on receiver operating characteristic (ROC) curve analysis (upper left point of the graph, showing the value with the best compromise between sensitivity and specificity) [13] (S2 Table). A binary logistic regression analysis was performed to measure the relationship between the categorial variables associated with the presence or absence of SARS-Cov-2 infection and the potential independent variables. Variables with non-statistical significance in the multivariate models were assessed to determine if they should be included in the final model because of their clinical importance. Odds-Ratio (OR) were used to assign points for the pre-test probability score, 2 points being assigned to the highest values (OR>10). The Hosmer-Lemeshow goodness of fit statistic [14] and Nagelkerke’s R^2^ were used to assess the model validity [15]. We performed a second ROC curve analysis to determine the area under the curve (AUC) and to choose the cutoff values, in order to determine the low-probability group (<5% probability of SARS-Cov-2 infection), the intermediate probability-group and the high-probability group (>90% of probability of SARS-Cov-2 infection). The proportion of patients with SARS-Cov-2 infection in each group was evaluated to check the accuracy of the PARIS score.

We assessed the ability of the score to differentiate patients and controls in the validation cohort by a ROC curve analysis and computed the ROC curve. The performance of the test according to the time period (with various prevalence of SARS-Cov-2 infection) was evaluated.

SPSS software (version 24, Chicago, IL, USA) was used for statistical analysis.

## Results

### Derivation cohort

**Fig 1:**
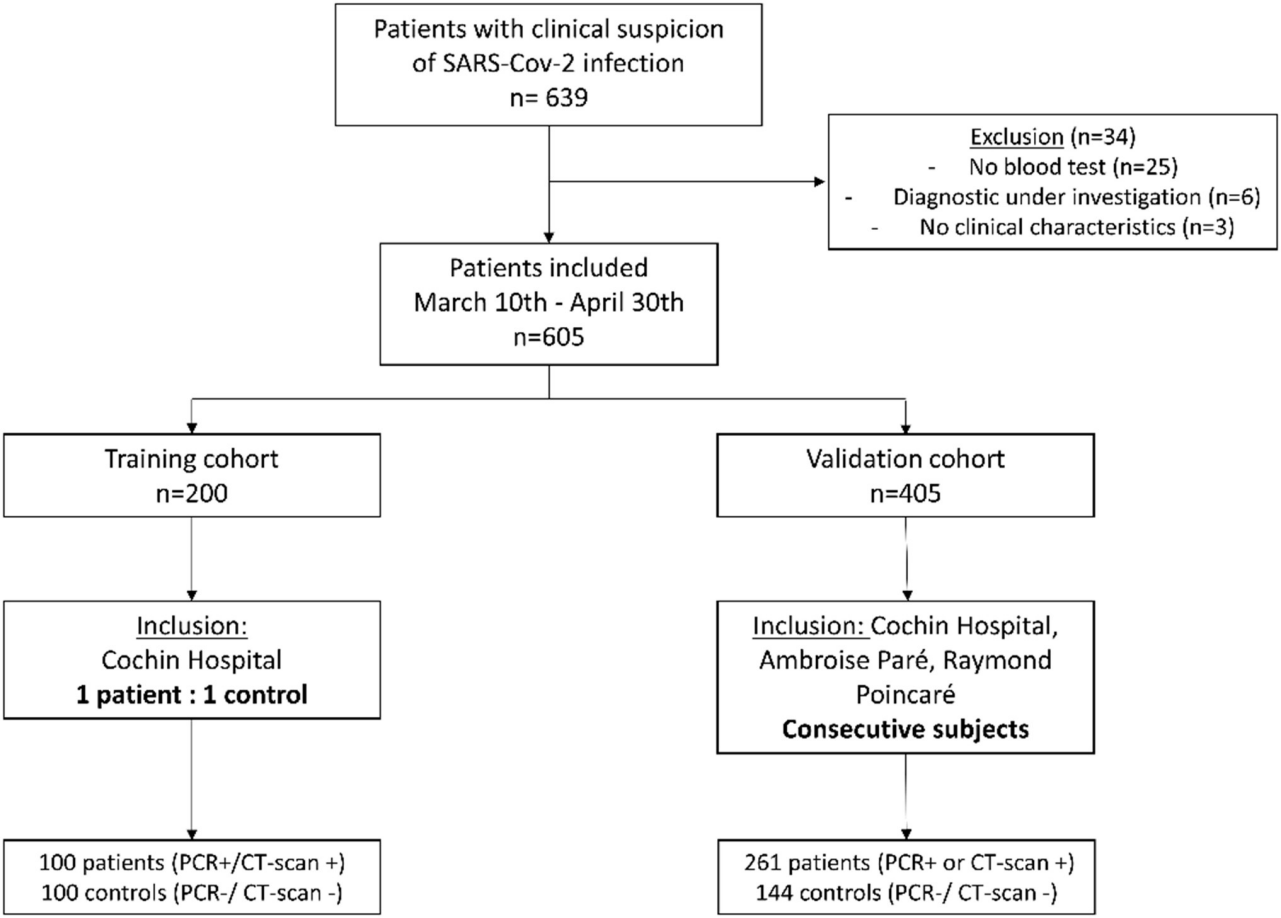
Flow chart of patients included.

The flow-chart of included patients is shown in Fig 1. The clinical and biological characteristics of the patients and controls included in the derivation cohort are presented in Table 1.

**Table 1:** Characteristics of patients with SARS-CoV-2 infection (confirmed with both PCR and CT) and controls.

	Patients	Controls	p-value
n	100	100	
Age	65 [24]	60 [31]	0.14
Sex (M:F)	65:35	45:55	0.005
*Clinical characteristics*			
Cough	79	66	0.04
Fever	90	63	<0.001
Temperature at ER	37.7 [1.4]	37 [0.7]	0.28
Shortness of breath	70	69	0.88
Saturation	95% [6]	96% [4]	0.03
Diarrhea	22	14	0.14
Myalgia	34	17	0.006
Headaches	16	18	0.71
Anosmia	11	5	0.12
Agueusia	14	4	0.01
Time from onset	6d [5]	5d [5]	0.12
*Comorbidities*			
Comorbidities	61	70	0.18
Diabetes	19	19	1
Hypertension	32	21	0.08
Renal failure	7	6	0.77
Pulmonary disease¤	17	43	<0.001
Immunodeficiency/ autoimmune disease	13	23	0.07
*Including*: *HIV*	2	5	0.25
*Treatments*			
Steroids	4	7	0.35
Chemotherapy	2	3	0.65
*Blood tests*			
Hemoglobin (g/dL)	13.70 [2.50]	13.20 [2.10]	0.31
Lymphocytes (G/L)	0.87 [0.48]	1.96. [1.18]	<0.001
Neutrophils (G/L)	4.29 [3.06]	6.47 [4.55]	<0.001
Eosinophils (G/L)	0.00 [0.01]	0.10 [0.19]	<0.001
Basophils (G/L)	0.01 [0.03]	0.04 [0.01]	<0.001
Monocytes (G/L)	0.44 [0.33]	0.70 [0.46]	<0.001
Platelets (G/L)	195.00 [91.75]	253.00 97.25]	<0.001
Sodium (mmol/L)	136.00 [5.00]	137.00 [4.25]	0.008
Potassium (mmol/L)	4.0 [0.50]	4.00 [0.40]	0.97
Chloride (mmol/L)	97.00 [4.00]	98.50 [5.00]	0.001
Bicarbonate (mmol/L)	24.00 [3.83]	24.00 [4.03]	0.82
Total protein (g/L)	71.80 [6.80]	74.00 [7.00]	0.07
Creatinine (μmol/L)	82.00 [33.50]	78.00 [31.50]	0.39
CRP (mg/L)	62.2 [78.20]	11.1 [67.40]	<0.001

^¤^Pulmonary disease: asthma, COPD or restrictive syndrome; Results are presented as median [interquartile range]; G/L = 10^9^/ Liter.

Fever, cough, oxygen saturation, agueusia and myalgias were the clinical variables that significantly differed between patients with confirmed SARS-Cov2 infection and controls in the univariate analysis. Patients with confirmed SARS-Cov2 infection had less frequent underlying pulmonary diseases (asthma, COPD or restrictive lung disease). Four percent of patients and 7% of controls were treated with corticosteroids. Among blood test variables, we noted significantly lower lymphocyte, neutrophil, eosinophil, monocyte, and basophil cell counts in patients as compared to controls. Additionally, the platelet count was significantly lower in patients with SARS-Cov-2 infection. Sodium, chlorides and CRP were significantly higher in patients.

All these variables were included in the multivariate binary logistic regression analysis. Only low lymphocyte, basophil, eosinophil, and neutrophil cell counts were significantly associated with SARS-Cov-2 infection (Table 2). The clinical variables were statistically insignificant and the performance of the score decreased when the clinical variables were included, perhaps due to inclusion of study participants with similar presenting symptoms in both cases and controls. ROC curve analysis determined the best cut-offs for sensitivity and specificity (S1 Table).

**Table 2:** Binary logistic regression using descending wald model.

	Patients	Controls	B	Exp(B)/OR	p-value
Basophils<0.04G/L	92	41	1.89	6.59	0.001
Eosinophils <0.06G/L	91	36	1.92	6.81	0.001
Lymphocytes<1.3G/L	89	24	2.56	12.88	<0.001
Neutrophils<5G/L	64	30	2.00	7.73	<0.001

OR: Odd-Ratio; Nagelkerke R^2^ = 0.70; Hosmer-Lemeshow goodness of fit statistic: p = 0.83.

Points were assigned according to the odds-ratio (Exp (B)): only lymphocytopenia (<1.3G/L) was associated with OR>10 (12.9) and 2 points were consequently assigned. The final score is presented in Table 3. AUC of the score in the derivation cohort was AUC = 0·924; STD = 0·019; CI = [0·887–0·961]. The Nagelkerke R^2^ was 0.70 and the Hosmer-Lemeshow goodness of fit statistic showed a p = 0.83.

**Table 3:** Pre-test diagnostic probability of COVID-19 infection: PARIS score.

Variables	Points
Eosinophils < 0.06 G/L	1
Lymphocytes < 1.3 G/L	2
Neutrophils <5G/L	1
Basophils <0.04G/L	1
Score = 0–1 → Low probability	
Score = 2–3 →Intermediate probability	
Score ≥4 → High probability	

### Validation cohort

**Fig 2:**
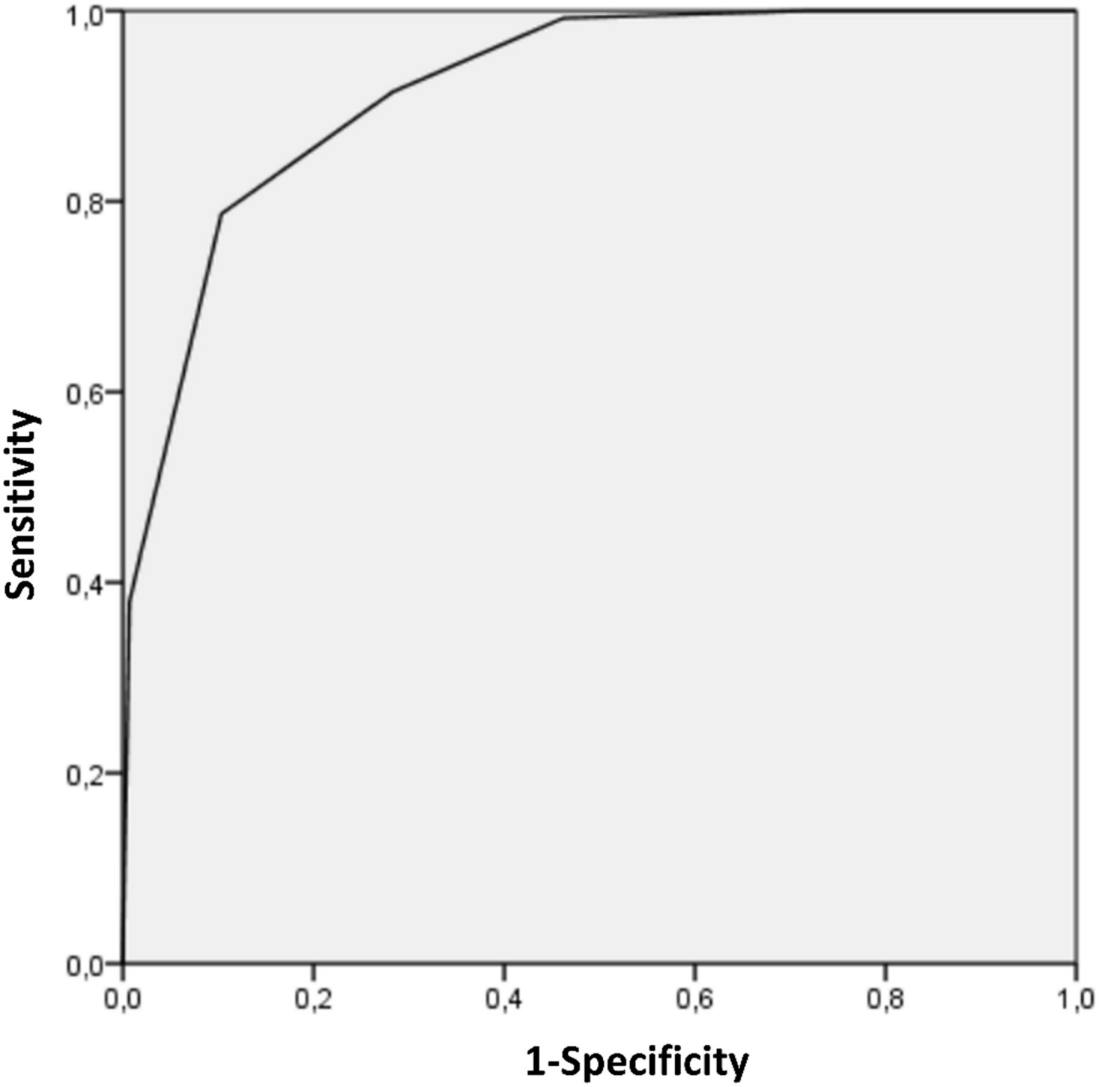
Receiver operating characteristic (ROC) curve of the PARIS score for the validation cohort. Area under the curve = 0.919.

Individuals included in the validation cohort had a mean age of 65 years (significantly older than those of the training cohort; p = 0·007), with 223 of male and 182 of female gender. There were 261 confirmed SARS-Cov-2 infected patients (64%) and 144 controls (36%). The pre-test probability score had a good performance in this cohort (AUC = 0·918 (CI: [0·891–0·946]; STD = 0·014). The sensitivity and specificity of the high-probability score (score 4 or 5) were 79% and 90% respectively (Table 4). Furthermore, the Negative Predictive Value (NPV) of a low-probability score (score 0 or 1) was 98% (CI = 0.93–0.99). A low-probability score was found for 54% of controls (N = 78/144). Many controls (36%) presented with intermediate probability scores (score 2 to 4) but the NPV of combined low- and intermediate-probability scores was only 70% (CI = 0.63–0.77). Fifty-three of the 261 SARS-Cov-2 infected patients (20%) from the cohort of validation had an intermediate score. No patient with SARS-Cov-2 infection had a score equal to 0 and there were only two infected patient with a score of 1. ROC curve for the validation cohort is presented in Fig 2.

**Table 4:** Performance in the validation cohort depending on the value of the PARIS score (performance for a score ≥ to the value).

PARIS score	Sensitivity	Specificity	PPV	NPV	N
**0**	**1**	**0**	**0.64**	**NA**	**40**
1	1	0.28	0.71	1	39
2	0.99	0.53	0.79	0.98	46
3	0.92	0.72	0.85	0.83	60
4	0.79	0.90	0.93	0.70	120
5	0.38	0.99	0.99	0.47	100

PPV = Positive Predictive Value; NPV = Negative Predictive Value; N: Number of Patients.

### Misclassification and reclassification

Twenty-nine controls (training and validation cohorts) presented with a high-probability score (≥4). The explanation for their lymphocytopenia was a septic shock (N = 3), immunodepression induced by autoimmune diseases and/or their treatments (N = 5), cystic fibrosis with lung transplantation (N = 2), pancytopenia due to hematological disorder under chemotherapy (N = 5), typhoid fever (N = 1) (S1 Table). Thirteen controls didn’t have any final diagnosis accounting for their lymphocytopenia and clinical symptoms. Only two patients with a low-probability score of 1 were considered as false-negatives of the pre-test probability score. These two patients had a negative RT-PCR result but positive CT-scan with minimal extent of pneumonia on CT (small ground glass opacities).

The score was also helpful to reclassify patients: Fourteen out of 21 infected patients, based on RT-PCR, with negative or indeterminate CT-scan results had a high-probability score (while the 7 other patients had an intermediate PARIS score). Twenty-one out of 31 patients with only negative PCR (1 to 3 tests) but CT features typical for COVID-19 pneumonia had a high-probability score (8 other patients had an intermediate score and 2 patients had a low score).

### Correlation between the PARIS score and CT-scan extent

The correlation between the PARIS score and the extent of the COVID-19 pneumonia on CT-scan is presented in the S3 Table. The proportion of patients with a high probability score was higher in patients having more than 10% of diseased lung parenchyma on CT while the proportion of patients with an intermediate probability score was higher in patients with minimal or no involvement on CT.

### Comparison of performance according to the incidence of the SARS-CoV-2 infections

The performance of the score was stable during the different time periods of the study with AUC ranging from 0.92 to 0.93, even if the proportion of patients and controls markedly changed over time (S4 Table). The prevalence of infection in the included patients was 68% between March 20^th^ and March 31^st^ versus only 31% between April 15^th^ and April 30^th^.

## Discussion

In this study, we established a pre-test probability score (PARIS score) based on simple, affordable, and widely available blood tests (complete white blood cell count). No clinical variable was independently associated with SARS-Cov-2 infection and thus included in this score but four biological variables (lymphocytes<1.3 G/L, eosinophils<0.06 G/L, basophils<0.04 G/L, neutrophils<5 G/L) were associated to the condition. This finding could be particularly useful for countries with a low availability of diagnostic tests. However, this score would need to be validated in other countries due to potential differences in populations, such as different ethnicity/age distributions compared to the population studied.

A low lymphocyte count has been described in patients with SARS-Cov-2 infection, possibly due to a decreased level of T-lymphocytes [16]. Furthermore, the total number of Natural Killer and CD8^+^ T cells has been reported to be markedly decreased in patients with SARS-Cov-2 infection [17]. The decrease of the absolute number of T lymphocytes is more pronounced in severe cases whereas decreased CD8^+^ T cells count could be an independent variable associated with the severity of the disease [18–20]. Eosinophils have also been reported to be decreased in previous studies [21, 22]. An increase of the eosinophil count may even be a predictor of improvement in patients with SARS-Cov-2 infection [23]. Even though the eosinopenia associated with COVID-19 is likely to be a secondary phenomenon not directly contributing to the disease course, its mechanism is not yet completely understood [24]. A low proportion of basophils has also been reported in severe cases [16] but no previous study has reported low basophil cell count as a marker of SARS-Cov-2 infection. Furthermore, previous reports demonstrated low levels of neutrophils in SARS-Cov-2 patients [25] but increased neutrophil count in severe cases of COVID-19 pneumonia [26]. Two previous histological studies performed on autopsies of deceased COVID-19 patients showed inflammatory cells including lymphocytes and macrophages in the alveoli, with minimal eosinophilic and neutrophilic infiltration [27, 28]. The decrease in the level of circulating lymphocytes could be due to their mobilization in the pulmonary infiltrates.

The Fleischner Society recently released a report highlighting to the lack of pre-test probability score in patients with mild symptoms consistent with SARS-Cov-2 infection [8]. The probability to be infected is mainly based on background prevalence of the disease and individual’s exposure risk. Committee members disapproved imaging use in case of mild symptoms and negative RT-PCR results. The PARIS score may enhance the proposed Fleischner diagnostic algorithm by improving the pre-test probability. Thus, in case of a high pre-test probability with no comorbidities explaining leukopenia, a second RT-PCR test or an additional CT-scan may be useful to establish the final diagnosis. Furthermore, a low PARIS score may obviate the need for RT-PCR or CT-scan, especially in a period of diagnostic test shortage. Chest radiography is insensitive in mild or early COVID-19 infection [8] but could be useful in combination of PARIS score in a resource-constrained environment.

A previous review indicated that prediction models for diagnosis and prognosis of COVID-19 infection are poorly reported, at high risk of bias, and with probably too optimistic reported performance [29]. We report here a multicenter study assessing diagnostic pre-test probability with a good accuracy, using a study design similar to that of the revised Geneve Score for pulmonary embolism [12].

Clinical symptoms were not included in our model, since most of covid-19 infected patients and controls presented with the same symptomatology, with no specificity of symptoms. Even oxygen saturation was not a discriminative feature. It could be explained by the fact that most patients with SARS-Cov2 infection were not severe, with subnormal oxygen saturation. Furthermore, even if patients with underlying lung diseases were more frequent in the control group, their average oxygen saturation was 96%. Thus, low oxygen saturation may be an additional argument for SARS-Cov-2 infection in patients with no previously diagnosed pulmonary disease. However, the PARIS score appears mostly useful in patients with relatively mild clinical presentation. It could also be more largely used for initial triage of patients presenting at the ED because it is fast, affordable and allows patients with a low probability score to stay apart from those most likely to be infected, avoiding the risk of contamination.

Our study presents inherent limitations. First, its retrospective design may introduce a selection bias. We chose to only include 100 patients and 100 controls in the training cohort in order to not delay the score development, so that it could be used in our hospitals as soon as possible during the pandemic. The proportion of controls in the validation cohort was lower, potentially affecting the estimation of the score predictive values. However, the confidence interval for the NPV of a low-probability score remained narrow in the validation cohort, despite this potential limitation. There were no statistically significant differences between demographic characteristics of patients with SARS-Cov-2 infection and controls but the 2 groups were not matched and controls more frequently presented pulmonary diseases and immunodeficiencies. We used only regular blood tests with complete white blood cell count, serum electrolytes and CRP, and did not evaluate LDH or D-dimers, since they seem to be mainly correlated with severity and have been described as risk factors for acute respiratory distress syndrome [30]. Another limitation of this study is the only inclusion of patients from hospital wards, which probably induces a recruitment bias, since they may not be representative of patients with milder symptoms who consult their general practitioner. The evaluation of the performance of our pre-test probability score in a larger population would be of interest. Even though a proportion of patients in our cohort were admitted to Intensive Care Units (ICUs) shortly after presentation, more severe patients directly admitted in ICUs, may be underrepresented. However, the score might not be as useful for severe patients admitted to ICU, since they anyway require admission and urgent care, regardless of RT-PCR positivity. This score was also developed during a pandemic with high prevalence of the disease and might not be as efficient in a situation where SARS-Cov2 infection would be rare, but its diagnostic performance remained stable even when prevalence of infection decreased in April. Numerous diseases or conditions may mimic SARS-Cov-2 biological changes with leukopenia and lymphocytopenia. Clinicians should be aware of the limits of this score due to other causes of variation of the white blood cell count. Lymphocyte count may be high in SARS-Cov-2 patients with leukemia or splenectomy [31, 32]. Conversely, sepsis, myelodysplasia, HIV infection, systemic lupus erythematous but also medications may induce lymphocytopenia in controls leading to a high-probability score [33, 34]. Lymphocytopenia is also a common finding in elderly patients [35], with increased difficulty of ruling out COVID-19 pneumonia in this population based on PARIS score. Another limitation is that most controls did not have a search for other respiratory viruses including influenza. Influenza can also induce lymphocytopenia [36, 37] with potential false positives of the score, but eosinopenia is less frequent in patients with influenza infection [38]. Low eosinophil counts is often related to the use of glucosteroids [39]. Eosinopenia is also considered as a surrogate of inflammation in bacterial infections [40] or myocardial infarction [41]. Basophil counts could be reduced by chronic urticaria or with steroids [42].

In conclusion, the PARIS score shows good performance with a high sensitivity and positive predictive value of intermediate and high probability scores and a high negative predictive value in low probability score individuals. It may help adapt the testing of subjects, especially during a pandemic with difficulties for large scale testing. Furthermore, future studies to evaluate the performance of the combination of PCR/CT and PARIS score could be of interest, in particular to detect COVID-19 patients with negative tests, that play a role in the diffusion of the virus.

## Supporting information

S1 Table Diagnoses of patients with clinical suspicion of SARS-Cov-2 infection and negative results (“= control cases”); COPD: Chronic obstructive pulmonary disease; GERD: Gastroesophageal reflux disease; * PCR tests of other viruses than SARS-Cov-2 (Influenza, Rhinovirus…) were only performed in 10 of the 144 controls. (DOCX)

S2 Table Receiver operator characteristic (ROC) curve analyses to determine the adequate cut-off for the independent variables; AUC = Area under the curve. (DOCX)

S3 Table PARIS score compared to CT-scan involvement in the 361 patients with SARS-Cov2 infection (including patients from both the training and validation cohorts). (DOCX)

S4 Table Performance of the PARIS score in the validation cohort according to the period of time (with increase, peak and decrease of virus diffusion among the population). (DOCX)

S5 Table Data of included patients in the derivation cohort (used to perform the ROC curve analysis, binary logistic regression analysis and the performance of the PARIS score in this cohort). (DOCX)
