# Supplementary material for: Pre-test probability for SARS-Cov-2-related infection score: The PARIS score
Source: PLoS One. 2020 Dec 17;15(12):e0243342. doi: 10.1371/journal.pone.0243342 (PMC7745977; doi:10.1371/journal.pone.0243342)
Supplement: S1 Table — (DOCX) [file pone.0243342.s001.docx]

**S1 Table.** Diagnoses of patients with clinical suspicion of SARS-Cov-2 infection and negative results (“=control cases”); COPD: chronic obstructive pulmonary disease; GERD: gastroesophageal reflux disease; * PCR tests of other viruses than SARS-Cov-2 (Influenza, Rhinovirus…) were only performed in 10 of the 144 controls

| **Diagnoses in controls** | **Training cohort (N=100)** | **Validation cohort (N=144)** |
| --- | --- | --- |
| No formal diagnosis | 26 | 38 |
| Asthma attack | 9 | 12 |
| COPD exacerbation | 10 | 18 |
| Bacterial pneumonia | 19 | 23 |
| Acute Pulmonary Edema | 6 | 12 |
| Sepsis | 5 | 7 |
| Pulmonary Embolism | 2 | 2 |
| Bronchitis | 7 | 7 |
| Typhoid fever | 1 | 0 |
| Lung cancer/metastasis | 4 | 5 |
| Arrhythmia | 2 | 0 |
| Pneumomediastinum | 1 | 0 |
| Pericarditis | 1 | 0 |
| Radiation-induced pneumonia | 1 | 0 |
| Adrenal insufficiency | 1 | 0 |
| Autoimmune disease | 2 | 3 |
| Pyelonephritis | 1 | 3 |
| GERD | 1 | 2 |
| Non-specific interstitial pneumonia | 0 | 2 |
| Flu* | 1 | 0 |
| Parvovirus | 0 | 1 |
| Diverticulitis | 0 | 2 |
| Anemia | 0 | 2 |
| Fungal pneumonia | 0 | 1 |
| Mononucleosis | 0 | 1 |
| Pneumocystis pneumonia | 0 | 1 |
| Heart attack | 0 | 1 |
| Zoster | 0 | 1 |
