# Supplementary material for: Pre-test probability for SARS-Cov-2-related infection score: The PARIS score
Source: PLoS One. 2020 Dec 17;15(12):e0243342. doi: 10.1371/journal.pone.0243342 (PMC7745977; doi:10.1371/journal.pone.0243342)
Supplement: S2 Table — (DOCX) [file pone.0243342.s002.docx]

**S2 Table.** Receiver Operator characteristic (ROC) curve analyses to determine the adequate cut-off for the independent variables; AUC= Area Under the Curve

| **Value (G/L)** | **Sensitivity** | **Specificity** | **AUC** |
| --- | --- | --- | --- |
| *Lymphocytes* | | | |
| 0.3 | 0.06 | 0.98 | 0.85 |
| 0.5 | 0.18 | 0.93 |  |
| 0.7 | 0.34 | 0.92 |  |
| 0.9 | 0.53 | 0.87 |  |
| 1.1 | 0.78 | 0.80 |  |
| **1.3** | **0.89** | **0.75** |  |
| 1.5 | 0.91 | 0.64 |  |
| 1.7 | 0.94 | 0.56 |  |
| 1.9 | 0.96 | 0.50 |  |
| 2.1 | 0.98 | 0.43 |  |
| *Eosinophils* | | | |
| 0.01 | 0.56 | 0.85 | 0.83 |
| 0.02 | 0.77 | 0.79 |  |
| 0.03 | 0.84 | 0.74 |  |
| 0.04 | 0.87 | 0.69 |  |
| 0.05 | 0.88 | 0.66 |  |
| **0.06** | **0.91** | **0.64** |  |
| 0.07 | 0.91 | 0.61 |  |
| 0.08 | 0.94 | 0.54 |  |
| 0.09 | 0.94 | 0.52 |  |
| 0.10 | 0.95 | 0.50 |  |
| *Basophils* | | | |
| 0.01 | 0.19 | 0.96 | 0.85 |
| 0.02 | 0.60 | 0.90 |  |
| 0.03 | 0.78 | 0.79 |  |
| **0.04** | **0.92** | **0.62** |  |
| 0.05 | 0.96 | 0.44 |  |
| 0.06 | 0.99 | 0.27 |  |
| *Neutrophils* | | | |
| 2 | 0.08 | 0.95 | 0.69 |
| 3 | 0.25 | 0.91 |  |
| 4 | 0.46 | 0.81 |  |
| **5** | **0.64** | **0.70** |  |
| 6 | 0.75 | 0.58 |  |
| 7 | 0.80 | 0.44 |  |
| 8 | 0.82 | 0.36 |  |
