# Supplementary material for: Pre-test probability for SARS-Cov-2-related infection score: The PARIS score
Source: PLoS One. 2020 Dec 17;15(12):e0243342. doi: 10.1371/journal.pone.0243342 (PMC7745977; doi:10.1371/journal.pone.0243342)
Supplement: S3 Table — (DOCX) [file pone.0243342.s003.docx]

**S3 Table**. PARIS score compared to CT-scan involvement in the 361 patients with SARS-Cov2 infection (including patients from both the training and validation cohorts).

| PARIS Score | None (N=22) | Minimal (<10%) (N=79) | Moderate (10-25%) (N=134) | Extended (25-50%) (N=98) | Severe (50-75%) (N=25) | Critical (>75%) (N=4) |
| --- | --- | --- | --- | --- | --- | --- |
| 0 | 0% | 0% | 0% | 0% | 0% | 0% |
| 1 | 0% | 2% | 0% | 0% | 0% | 0% |
| 2 | 23% | 20% | 3% | 9% | 0% | 0% |
| 3 | 15% | 20% | 20% | 13% | 15% | 0% |
| 4 | 8% | 30% | 34% | 41% | 46% | 100% |
| 5 | 54% | 28% | 43% | 37% | 39% | 0% |
