# Supplementary material for: Pre-test probability for SARS-Cov-2-related infection score: The PARIS score
Source: PLoS One. 2020 Dec 17;15(12):e0243342. doi: 10.1371/journal.pone.0243342 (PMC7745977; doi:10.1371/journal.pone.0243342)
Supplement: S4 Table — (DOCX) [file pone.0243342.s004.docx]

**S4 Table.** Performance of the PARIS score in the validation cohort according to the period of time (with increase, peak and decrease of virus diffusion among the population)

|  | March 10^th^-19^th^ | March 20^th^- 31^st^ | April 1^st^-15^th^ | April 16^th^-30^th^ |
| --- | --- | --- | --- | --- |
| Patients | 73 | 160 | 97 | 29 |
| Controls | 39 | 75 | 67 | 65 |
| AUC | 0.92 | 0.93 | 0.92 | 0.92 |
| *Low-score (0-1)* | | | | |
| Sensitivity | 1 | 1 | 1 | 1 |
| Specificity | 0.56 | 0.57 | 0.54 | 0.55 |
| PPV | 0.65 | 0.68 | 0.59 | 0.31 |
| NPV | 1 | 0.98 | 1 | 0.97 |
| *High-score (4-5)* | | | | |
| Sensitivity | 0.81 | 0.82 | 0.74 | 0.86 |
| Specificity | 0.85 | 0.89 | 0.90 | 0.88 |
| PPV | 0.91 | 0.94 | 0.91 | 0.76 |
| NPV | 0.70 | 0.70 | 0.71 | 0.93 |
