# Supplementary material for: Pre-test probability for SARS-Cov-2-related infection score: The PARIS score
Source: PLoS One. 2020 Dec 17;15(12):e0243342. doi: 10.1371/journal.pone.0243342 (PMC7745977; doi:10.1371/journal.pone.0243342)
Supplement: S5 Table — (DOCX) [file pone.0243342.s005.docx]

**S5 Table**. Data of included patients in the derivation cohort.

| **Patient** | **Lympho** | **Baso** | **Eosino** | **Neutro** | **CRP** | **Platelets** | **COVID** |
| --- | --- | --- | --- | --- | --- | --- | --- |
| 1 | 1,080 | ,010 | ,010 | 10,8 | 38,9 | 188 | Yes |
| 2 | ,360 | ,020 | ,000 | 8,9 | 22,6 | 132 | Yes |
| 3 | ,280 | ,030 | ,000 | 4,5 | NA | 283 | Yes |
| 4 | ,740 | ,030 | ,000 | 1,8 | 107,2 | 257 | Yes |
| 5 | 1,980 | ,010 | ,130 | 4,5 | 27,9 | 582 | Yes |
| 6 | 1,120 | ,000 | ,070 | 4,6 | 19,6 | 318 | Yes |
| 7 | ,270 | ,010 | ,000 | 3,0 | 132,0 | 172 | Yes |
| 8 | ,600 | ,000 | ,000 | 8,7 | 140,0 | 320 | Yes |
| 9 | ,620 | ,020 | ,000 | 3,4 | 72,0 | 176 | Yes |
| 10 | ,240 | ,010 | ,000 | 1,6 | 38,4 | 280 | Yes |
| 11 | ,990 | ,020 | ,000 | 2,8 | 65,0 | 179 | Yes |
| 12 | ,840 | ,030 | ,000 | 2,8 | 95,0 | 110 | Yes |
| 13 | ,720 | ,000 | ,020 | 1,8 | NA | 109 | Yes |
| 14 | 2,010 | ,010 | ,010 | 2,7 | NA | 306 | Yes |
| 15 | ,630 | ,040 | ,010 | 6,5 | 62,6 | 121 | Yes |
| 16 | 1,090 | ,020 | ,020 | 5,8 | 24,0 | 192 | Yes |
| 17 | 1,090 | ,050 | ,010 | 2,7 | 3,4 | 237 | Yes |
| 18 | ,890 | ,040 | ,000 | 6,9 | 147,0 | 158 | Yes |
| 19 | ,980 | ,030 | ,050 | 4,6 | 12,6 | 132 | Yes |
| 20 | 1,100 | ,050 | ,670 | 10,4 | 85,7 | 175 | Yes |
| 21 | ,860 | ,000 | ,070 | 8,3 | 13,1 | 204 | Yes |
| 22 | 1,110 | ,010 | ,050 | 8,9 | 56,5 | 211 | Yes |
| 23 | ,930 | ,020 | ,000 | 4,0 | 117,2 | 325 | Yes |
| 24 | ,330 | ,010 | ,010 | 2,9 | 150,8 | 429 | Yes |
| 25 | 1,840 | ,020 | ,010 | 2,1 | 11,3 | 161 | Yes |
| 26 | ,870 | ,010 | ,000 | 4,6 | 5,1 | 117 | Yes |
| 27 | ,590 | ,010 | ,010 | 1,0 | 23,3 | 184 | Yes |
| 28 | ,930 | ,010 | ,020 | 3,9 | NA | 180 | Yes |
| 29 | ,360 | ,030 | ,010 | 3,5 | 39,0 | 90 | Yes |
| 30 | ,600 | ,010 | ,010 | 4,3 | 106,7 | 175 | Yes |
| 31 | ,790 | ,020 | ,140 | 3,9 | 4,1 | 124 | Yes |
| 32 | ,990 | ,000 | ,000 | 3,4 | 10,0 | 162 | Yes |
| 33 | 1,090 | ,030 | ,010 | 2,5 | 80,3 | 209 | Yes |
| 34 | ,650 | ,000 | ,000 | 4,0 | 44,0 | 199 | Yes |
| 35 | 2,620 | ,030 | ,150 | 12,2 | 7,2 | 153 | Yes |
| 36 | ,650 | ,010 | ,000 | 4,3 | 69,1 | 146 | Yes |
| 37 | ,350 | ,000 | ,000 | 1,6 | 170,8 | 307 | Yes |
| 38 | 1,340 | ,030 | ,000 | 2,7 | 115,2 | 157 | Yes |
| 39 | ,860 | ,010 | ,000 | 6,7 | 50,9 | 111 | Yes |
| 40 | 1,550 | ,010 | ,000 | 2,9 | NA | 178 | Yes |
| 41 | ,730 | ,010 | ,010 | 1,0 | 44,0 | 250 | Yes |
| 42 | 1,090 | ,010 | ,000 | 2,8 | 35,0 | 207 | Yes |
| 43 | ,820 | ,020 | ,000 | 7,6 | 109,6 | 103 | Yes |
| 44 | 1,280 | ,010 | ,010 | 3,5 | 25,7 | 193 | Yes |
| 45 | 1,230 | ,010 | ,000 | 3,0 | 22,6 | 231 | Yes |
| 46 | 1,200 | ,020 | ,020 | 9,1 | 35,6 | 222 | Yes |
| 47 | ,850 | ,010 | ,000 | 3,7 | 51,2 | 215 | Yes |
| 48 | ,980 | ,010 | ,020 | 4,6 | 25,7 | 319 | Yes |
| 49 | ,670 | ,000 | ,010 | 4,9 | 31,3 | 102 | Yes |
| 50 | ,470 | ,040 | ,000 | 6,8 | 97,4 | 276 | Yes |
| 51 | 1,280 | ,010 | ,000 | 9,9 | 44,6 | 291 | Yes |
| 52 | ,610 | ,020 | ,010 | 5,9 | 49,2 | 192 | Yes |
| 53 | ,390 | ,040 | ,000 | 4,4 | 61,8 | 353 | Yes |
| 54 | ,510 | ,010 | ,000 | 5,2 | 117,6 | 200 | Yes |
| 55 | ,940 | ,000 | ,090 | 3,3 | 2,2 | 219 | Yes |
| 56 | ,530 | ,010 | ,010 | 4,3 | 18,4 | 150 | Yes |
| 57 | ,560 | ,020 | ,010 | 3,5 | 37,0 | 197 | Yes |
| 58 | ,930 | ,020 | ,000 | 5,1 | NA | 196 | Yes |
| 59 | 1,520 | ,010 | ,010 | 1,6 | 33,3 | 199 | Yes |
| 60 | 1,040 | ,010 | ,000 | 3,5 | 54,2 | 244 | Yes |
| 61 | 1,050 | ,020 | ,030 | 3,3 | 116,6 | 200 | Yes |
| 62 | ,810 | ,030 | ,000 | 17,5 | 212,8 | 136 | Yes |
| 63 | 1,360 | ,020 | ,010 | 5,8 | 14,1 | 143 | Yes |
| 64 | 2,410 | ,030 | ,020 | 13,4 | 378,9 | 424 | Yes |
| 65 | ,780 | ,010 | ,000 | 3,2 | 105,8 | 198 | Yes |
| 66 | ,320 | ,010 | ,000 | 3,7 | 168,8 | 405 | Yes |
| 67 | 1,230 | ,030 | ,000 | 12,0 | 129,1 | 117 | Yes |
| 68 | ,910 | ,010 | ,000 | 3,7 | 76,4 | 77 | Yes |
| 69 | ,560 | ,000 | ,000 | 5,4 | 200,9 | 161 | Yes |
| 70 | ,520 | ,000 | ,010 | 14,3 | 52,1 | 201 | Yes |
| 71 | ,930 | ,000 | ,000 | 4,2 | 50,7 | 100 | Yes |
| 72 | ,260 | ,000 | ,000 | 4,4 | 102,8 | 116 | Yes |
| 73 | ,680 | ,010 | ,010 | 5,3 | 84,4 | 275 | Yes |
| 74 | ,470 | ,010 | ,000 | 2,2 | 67,2 | 123 | Yes |
| 75 | ,930 | ,030 | ,030 | 3,0 | 30,2 | 229 | Yes |
| 76 | ,100 | ,010 | ,000 | 12,7 | 105,8 | 98 | Yes |
| 77 | 1,610 | ,020 | ,150 | 2,9 | 1,2 | 293 | Yes |
| 78 | ,660 | ,030 | ,000 | 9,9 | 169,6 | 180 | Yes |
| 79 | ,950 | ,010 | ,050 | 7,5 | 126,0 | 99 | Yes |
| 80 | ,300 | ,020 | ,000 | 6,7 | 401,5 | 170 | Yes |
| 81 | ,690 | ,010 | ,000 | 5,6 | NA | 141 | Yes |
| 82 | ,970 | ,000 | ,000 | 1,9 | 79,8 | 203 | Yes |
| 83 | ,720 | ,000 | ,010 | 5,5 | 45,3 | 204 | Yes |
| 84 | ,370 | ,010 | ,000 | 5,0 | 144,9 | 197 | Yes |
| 85 | ,890 | ,020 | ,000 | 8,1 | 78,2 | 260 | Yes |
| 86 | 1,250 | ,030 | ,070 | 14,9 | 57,4 | 205 | Yes |
| 87 | ,730 | ,000 | ,000 | 4,0 | 155,1 | 244 | Yes |
| 88 | ,850 | ,010 | ,020 | 4,8 | 158,7 | 431 | Yes |
| 89 | ,960 | ,010 | ,000 | 5,8 | 94,1 | 170 | Yes |
| 90 | 1,120 | ,010 | ,000 | 3,6 | 143,7 | 162 | Yes |
| 91 | ,370 | ,010 | ,000 | 2,7 | 134,8 | 311 | Yes |
| 92 | 1,130 | ,000 | ,000 | 3,7 | 89,7 | 177 | Yes |
| 93 | 1,810 | ,010 | ,030 | 2,5 | 22,3 | 202 | Yes |
| 94 | 1,150 | ,000 | ,000 | 5,5 | 56,4 | 212 | Yes |
| 95 | ,930 | ,010 | ,000 | 3,4 | 59,8 | 158 | Yes |
| 96 | ,450 | ,000 | ,000 | 3,4 | 78,5 | 99 | Yes |
| 97 | ,940 | ,050 | ,000 | 4,3 | 68,0 | 162 | Yes |
| 98 | 1,300 | ,100 | ,040 | 10,7 | 49,6 | 267 | Yes |
| 99 | ,760 | ,050 | ,000 | 2,0 | NA | 98 | Yes |
| 100 | ,720 | ,060 | ,000 | 9,6 | 291,1 | 263 | Yes |
| 101 | 1,050 | ,020 | ,060 | 10,2 | 10,2 | 219 | No |
| 102 | 2,040 | ,100 | ,120 | 8,0 | NA | 229 | No |
| 103 | ,800 | ,040 | ,000 | 7,1 | 58,2 | 262 | No |
| 104 | 2,040 | ,010 | ,010 | 1,9 | 1,0 | 284 | No |
| 105 | 2,570 | ,050 | ,040 | 8,9 | NA | 257 | No |
| 106 | ,350 | ,050 | ,000 | 4,5 | 263,7 | 128 | No |
| 107 | 2,120 | ,050 | ,160 | 11,0 | 52,2 | 732 | No |
| 108 | 1,290 | ,050 | ,230 | 7,8 | NA | 256 | No |
| 109 | 1,350 | ,020 | ,030 | 4,0 | 66,0 | 231 | No |
| 110 | 1,720 | ,060 | ,120 | 2,2 | 113,5 | 155 | No |
| 111 | ,970 | ,020 | ,070 | 3,1 | 47,2 | 239 | No |
| 112 | 1,630 | ,050 | ,070 | 3,4 | 12,3 | 244 | No |
| 113 | 2,220 | ,010 | ,100 | 6,8 | NA | 268 | No |
| 114 | 2,710 | ,040 | ,220 | 4,6 | 5,0 | 238 | No |
| 115 | 1,140 | ,090 | ,030 | 3,6 | 18,2 | 89 | No |
| 116 | 2,370 | ,060 | ,100 | 2,2 | 3,1 | 201 | No |
| 117 | 2,930 | ,050 | ,180 | 9,9 | 1,0 | 208 | No |
| 118 | 2,970 | ,090 | ,030 | 9,0 | 18,9 | 158 | No |
| 119 | 2,450 | ,020 | ,580 | 7,8 | 53,6 | 293 | No |
| 120 | 2,100 | ,040 | ,260 | 6,1 | NA | 305 | No |
| 121 | 2,250 | ,000 | ,270 | 12,3 | 66,3 | 226 | No |
| 122 | 2,190 | ,030 | ,100 | 6,2 | 6,5 | 279 | No |
| 123 | 1,940 | ,050 | ,000 | 14,1 | 224,8 | 308 | No |
| 124 | ,870 | ,040 | ,400 | 6,8 | 55,6 | 285 | No |
| 125 | 1,740 | ,040 | ,100 | 5,7 | 206,0 | 554 | No |
| 126 | 1,660 | ,030 | ,270 | 8,6 | 4,0 | 226 | No |
| 127 | 1,630 | ,110 | ,370 | 7,7 | 67,7 | 233 | No |
| 128 | 1,310 | ,040 | ,000 | 4,0 | 113,8 | 176 | No |
| 129 | 2,070 | ,060 | ,570 | 2,3 | NA | 306 | No |
| 130 | 3,030 | ,040 | ,130 | 8,5 | 3,7 | 280 | No |
| 131 | 3,010 | ,020 | ,210 | 6,4 | 34,8 | 375 | No |
| 132 | 2,150 | ,210 | ,130 | 7,9 | 2,0 | 274 | No |
| 133 | 1,060 | ,040 | ,100 | 4,6 | 5,4 | 186 | No |
| 134 | 2,180 | ,020 | ,980 | 4,9 | 3,8 | 292 | No |
| 135 | 1,540 | ,070 | ,250 | 6,7 | 59,2 | 213 | No |
| 136 | 1,420 | ,000 | ,060 | 15,0 | 1,3 | 253 | No |
| 137 | 2,370 | ,100 | ,030 | 5,1 | 1,0 | 326 | No |
| 138 | 1,340 | ,060 | ,000 | 15,8 | 613,7 | 398 | No |
| 139 | 5,820 | ,000 | ,000 | 1,9 | 1,6 | 241 | No |
| 140 | 2,740 | ,050 | ,000 | 5,0 | 72,4 | 427 | No |
| 141 | ,450 | ,030 | ,000 | 3,8 | 6,6 | 186 | No |
| 142 | 2,460 | ,040 | ,270 | 5,0 | NA | 229 | No |
| 143 | 2,640 | ,060 | ,260 | 11,3 | NA | 275 | No |
| 144 | 1,900 | ,100 | ,150 | 19,1 | NA | 253 | No |
| 145 | 3,160 | ,160 | ,020 | 17,2 | NA | 202 | No |
| 146 | 2,270 | ,100 | 1,200 | 18,6 | 1,0 | 308 | No |
| 147 | 3,340 | ,050 | ,990 | 5,2 | 1,2 | 313 | No |
| 148 | 3,250 | ,050 | ,070 | 10,2 | 303,0 | 395 | No |
| 149 | 1,420 | ,020 | ,080 | 5,8 | 1,3 | 232 | No |
| 150 | 2,200 | ,030 | ,130 | 8,5 | 92,0 | 189 | No |
| 151 | 1,450 | ,030 | ,030 | 6,2 | 1,0 | 316 | No |
| 152 | 1,340 | ,020 | ,020 | 5,6 | 132,8 | 299 | No |
| 153 | 3,240 | ,060 | ,090 | 5,2 | 2,0 | 299 | No |
| 154 | 1,480 | ,060 | ,040 | 11,9 | 1,0 | 212 | No |
| 155 | 2,980 | ,120 | ,370 | 38,7 | 5,0 | 323 | No |
| 156 | 1,680 | ,030 | ,060 | 10,8 | 1,0 | 313 | No |
| 157 | ,810 | ,030 | ,000 | 9,1 | 271,2 | 343 | No |
| 158 | 1,520 | ,010 | ,010 | 5,7 | 1,0 | 278 | No |
| 159 | ,980 | ,090 | ,050 | 7,0 | 141,7 | 268 | No |
| 160 | ,210 | ,050 | ,000 | 6,2 | 34,4 | 196 | No |
| 161 | 2,570 | ,050 | ,210 | 4,5 | 1,0 | 297 | No |
| 162 | 2,100 | ,050 | ,100 | 4,4 | 21,0 | 381 | No |
| 163 | 2,240 | ,030 | ,300 | 9,9 | 1,0 | 313 | No |
| 164 | 2,400 | ,030 | ,120 | 10,6 | 3,0 | 293 | No |
| 165 | 2,820 | ,060 | ,240 | 5,6 | 1,0 | 328 | No |
| 166 | ,100 | ,020 | ,000 | 4,3 | 112,5 | 204 | No |
| 167 | 3,140 | ,040 | ,070 | 2,7 | 1,0 | 195 | No |
| 168 | 3,520 | ,050 | ,070 | 5,3 | 1,0 | 363 | No |
| 169 | 1,800 | ,030 | ,160 | 5,8 | 1,0 | 257 | No |
| 170 | 2,400 | ,010 | ,120 | 2,0 | 2,4 | 273 | No |
| 171 | 1,380 | ,040 | ,090 | 13,5 | 1,0 | 256 | No |
| 172 | ,350 | ,180 | ,010 | 21,8 | 3,0 | 85 | No |
| 173 | ,730 | ,080 | ,000 | 8,2 | 17,0 | 277 | No |
| 174 | 1,160 | ,060 | ,000 | 9,3 | 144,3 | 54 | No |
| 175 | 2,690 | ,040 | ,590 | 4,6 | 13,3 | 364 | No |
| 176 | 2,600 | ,090 | ,150 | 12,1 | 265,6 | 510 | No |
| 177 | 1,580 | ,040 | ,050 | 85,6 | 1,0 | 220 | No |
| 178 | 1,000 | ,000 | ,230 | 6,5 | NA | 174 | No |
| 179 | ,620 | ,030 | ,010 | 7,4 | 199,0 | 154 | No |
| 180 | 1,300 | ,040 | ,450 | 6,3 | 12,0 | 342 | No |
| 181 | 1,670 | ,030 | ,140 | 9,0 | 2,3 | 180 | No |
| 182 | 2,420 | ,030 | ,370 | 6,7 | 2,6 | 175 | No |
| 183 | 1,830 | ,040 | ,180 | 3,6 | 110,0 | 264 | No |
| 184 | 1,760 | ,030 | ,110 | 3,1 | NA | 177 | No |
| 185 | 3,020 | ,030 | ,170 | 13,0 | 6,9 | 203 | No |
| 186 | 2,210 | ,050 | ,120 | 1,5 | 1,0 | 217 | No |
| 187 | ,370 | ,030 | ,020 | 6,2 | 127,2 | 94 | No |
| 188 | 2,750 | ,040 | ,150 | 8,9 | 1,3 | 219 | No |
| 189 | 2,420 | ,010 | ,070 | 3,5 | 1,0 | 310 | No |
| 190 | 2,740 | ,010 | ,010 | 7,7 | 40,1 | 164 | No |
| 191 | 2,620 | ,020 | ,040 | 9,1 | 3,0 | 237 | No |
| 192 | 1,290 | ,030 | ,000 | 9,2 | 350,8 | 175 | No |
| 193 | ,510 | ,030 | ,020 | 3,8 | 123,6 | 159 | No |
| 194 | 6,910 | ,020 | ,020 | 5,7 | 249,3 | 201 | No |
| 195 | 1,970 | ,030 | ,070 | 9,1 | 8,0 | 213 | No |
| 196 | 1,310 | ,070 | ,120 | 4,5 | 28,2 | 272 | No |
| 197 | ,740 | ,090 | ,010 | 6,4 | 308,8 | 167 | No |
| 198 | 1,020 | ,080 | ,000 | 6,1 | 15,8 | 159 | No |
| 199 | 1,020 | ,010 | ,450 | 10,8 | 20,0 | 284 | No |
| 200 | 1,980 | ,020 | ,930 | 8,9 | 4,7 | 234 | No |

*Abbreviations* : Lympho= lymphocytes ; Baso= Basophils ; Eosino= Eosinophils ; Neutro= Neutrophils
